# A three-staged framework for measuring water supply resilience in rural China based on PLS-SEM

**DOI:** 10.1038/s41598-022-08112-4

**Published:** 2022-03-12

**Authors:** Wenmei Zhou, Gretchen Kalonji, Chuan Chen, Hongyan Zheng, Igor Martek

**Affiliations:** 1 The Hong Kong Polytechnic University Institute for Disaster Management and Reconstruction, Sichuan University, Chengdu, Sichuan China; 2 Business School, Sichuan University, Chengdu, Sichuan China; 3 School of Architecture and Built Environment, Deakin University, Geelong, VIC 3220 Australia

**Keywords:** Climate-change adaptation, Climate-change impacts, Natural hazards

## Abstract

China suffers from frequent large-scale earthquakes, posing a significant challenge to the development and integrity of its rural water supply system (RWSS). The earthquake resilience of water supply systems is understood to be a function of multifaceted factors, which are time- and space-dependent. Measuring the seismic-resilience of RWSS in China remains a challenge. This paper proposes a multi-stage comprehensive evaluation framework, focusing on the relationship between multi-dimensional factors and the seismic- resilience of RWSS in rural areas, across three stages: before, during and after earthquake events. This study comprises four steps: (1) Development of a multi-stage evaluation conceptual framework; (2) identification of seismic-resilience factors; (3) verification of the relationships between factors and stages; and (4) formation of the final evaluation framework. The relationship between multi-dimensional factors is confirmed by a method of triangulation through the quantitative analysis of PLS-SEM combined with the qualitative literature analysis, highlighting the causal approach of the resilience of RWSSs, so as to better understand the resilience state of each stage of disaster. Understanding these factors and their influence on the seismic capacity of RWSS will enable local authorities to recognize the existing advantages and disadvantages of these factors, so as to carry out better resilience practice in all stages of disasters.

## Introduction

Rural drinking water services in developing countries have been significantly improved with the promotion of the United Nations Millennium Development Goals and Sustainable Development Goals^1^. The Chinese government had heavily invested in the construction of RWSSs from 2016 to 2019, which improved the rate of centralized water supply in rural China from 68.7% to 86%^2^. The rural water supply infrastructure has been greatly improved in China. However, the frequent earthquakes in rural China have brought great damage to the rapid development of RWSSs, as shown in Table 1. Moreover, water shortage after the earthquake may bring secondary disasters and greater indirect losses just like the post-earthquake fire in Japan^3^ and the post-earthquake cholera epidemic in Haiti^4^. The water supply system provides vital services for the community^5^, and adequate water at an acceptable level of service in disasters must be provided^6,7^. Thus, the current seismic resilience of RWSSs based on the development of rural water supply infrastructure and earthquake history in China must be determined.

**Table 1:** Earthquake damage to RWSSs (adapted from Wenmei et al.^2^).

No	Magnitude	Year	Epicenter location	Consequences of damage to RWSSs	
				Destruction of Rural water supply project (place)	Number of people suffering water shortage
1	7.0	2013	Lushan County, Sichuan	1727	85,0000
2	7.1	2010	Yushu County, Qinghai	1123	82,800
3	8.0	2008	Wenchuan County, Sichuan	49,949	9,555,000

The main purpose of measuring system resilience is to understand resilience and its potential influencing factors to provide decision makers with appropriate information about the most vulnerable components of the system^6^. Identifying resilience indicators is the first step to measuring resilience. Resilience indicators enable different levels of administration to integrate resilience development strategies into mitigation and preparedness plans^6^. However, there is no widely accepted list of seismic-resilience measurement indicators of RWSSs, and the seismic-resilience of RWSS is difficult to evaluate.

Even if all the key factors to measure the seismic resilience of RWSSs are identified, a framework to deal with these key factors needs to be developed. The seismic resilience of water supply systems is affected by multi-dimensional factors, which may restrict and influence each other. Decision makers need to fully understand the relationship between these factors when evaluating the system resilience. Some studies on the seismic resilience of the water supply system have been conducted^6–8^, subjectively defining the relationship among factors. However, these studies did not objectively analyze the relationship among factors, and most of the research were mainly for the urban water supply system (UWSS)^9^. There is a lack of understanding of seismic resilience of RWSSs and the relationship among the driving factors due to the spatial differences in disaster resilience, and the individual drivers of which widely vary between rural and urban infrastructures^10^. Therefore, this study aims to establish the evaluation framework of RWSSs by exploring the relationship between driving factors. This goal will be achieved through four steps: (1) Development of a multi-stage evaluation conceptual framework; (2) identification of seismic-resilience factors; (3) verification of the relationships between factors and stages; and (4) formation of the final evaluation framework.

## Literature review

### Definition of infrastructure resilience

Holling^11^ was the first to refer to resilience in describing the capacity of natural ecosystems. More recently, the resilience of infrastructure has generated significant research interest as a consequence of the increased prevalence of natural disasters such as earthquakes^6,12–16^. Resilience, however, is a very broad concept. In evaluating the resilience of infrastructure, various studies have invoked a range of definitions. The Multidisciplinary Center for Earthquake Engineering Research *describes it as ‘‘the ability of the system to reduce the chances of shock, to absorb a shock if it occurs, and to recover quickly after a shock (re-establish normal performance)”*^8^. NIAC^17^ define infrastructure resilience as *“the ability to predict, absorb, adapt and/or quickly recover from destructive events such as natural disasters”.* The CSIRO, Australia^18^, state resilient infrastructure *“should have the ability of coordinated planning across departments and networks, be able to provide the lowest level of services during outages, emergencies and disasters, and quickly resume full operation.”* While these definitions vary, they share in common three characteristics: absorptive, adaptive, and restorative capacities^6,19^.

A major issue of debate regarding infrastructure resilience pertains to whether restoring the system to its pre-outage state is the most desirable outcome^9^. In considering resilience as a static state, emphasis is placed on returning the engineering capacity of the system to its pre-disaster status^20^, Studies of this nature focus on the physical vulnerability of infrastructure, and tend to assess the damage to infrastructure arising directly from disasters^21–23^ or the assets ability to recover in the post-disaster period^16^. In neither case are other dimensions nor other stages of the procurement cycle considered. Chandler and Coffee^24^ believe that a steady state (static concept) represents the first generation of resilience thinking. A second generation conceptualization of resilience, however, is more dynamic and encompasses growth and development^24^. When considering the important dynamic components of uncertainty that have erstwhile not been considered in previous literature, the concept of resilience begins to shift, becoming no longer just an inherent feature of a system, but an evolving process^9^. In this conceptualization, the restorative capacity of resilience tends to recover to a state better than existed pre-disaster, embracing new opportunities and understandings catalyzed by the post disaster reconstruction^25^.

### Resilience measurement of water supply systems

Early studies focused on assessing the indirect loss arising from interruptions to infrastructure operations, putting forward corresponding loss estimation models^26,27^. However, these models only evaluated economic loss, and overlooked any evaluation of the infrastructures capacity for resilience^28^. Bruneau et al.^8^ proposed the "TOSE" model, which evaluates the seismic resilience of communities and infrastructure, based on four dimensions. These are: technical, economic, organizational, and social, though stopping short of actual implementation. Based on the “TOSE” Model, Chang et al.^28^ developed an earthquake loss estimation model in order to quantify the technical and organizational resilience of water supply systems in Memphis, Tennessee, by simulating the loss caused by water supply system interruption under different earthquake magnitude scenarios. However, Chang et al.^28^ research focused on earthquake prevention and disaster reduction pre-disaster, and did not consider the impact of organizational and social responses on the maintenance of water supply services during the actual emergency response stage.

Time is critical factor when considering resilience as a process before, during, and after a disaster^9,24,29,30^. The concept of quantitatively evaluating infrastructure elasticity in accordance with change of system service level over time has been widely employed^31^. While some researchers have dynamically evaluated infrastructure resilience across three stages from a technical point of view^32,33^, it is important to note that resilience does not only depend on the physical vulnerability of the system but also has multidimensional characteristics^6,8,9,34^. Some work has been done in this area^6,34^. Balaei et al.^6^ proposed a "CARE" model based on the relationship between water supply service and time to evaluate the impact of technical, economic, environmental, organizational and social dimensions on the seismic resilience of water supply systems. While the “CARE” model emphasizes the time-varying characteristics of resilience, it emphasizes the resilience of the system in the post-earthquake period. In addition, the correlation of multi-dimensional factors is not reflected in the model. Nevertheless, these factors will not affect the post-disaster water supply alone, but are mutually influential^35^. As a key component of community resilience, a water infrastructure system with high resilience may fail in the future, and may not maintain performance at an acceptable level over the totality of its service life due to changing conditions^36^.

The latest research highlights the importance of the dynamic relationship between multi-dimensional factors^37^. Moreover, it considers resilience from the whole of disaster management cycle, given that communities can be understood to exist between the two disasters—the past and future one to come. In this conceptualization, resilience to past disasters impacts the resilience of communities to future disasters^25^.

In summary, resilience measurement is evolving towards a multi-dimensional and dynamic measurement regime, based on a comprehensive longitudinal disaster cycle (before, during, and after the disaster). However, Sharifi and Yagamata^34^, examining 36 resilience assessment tools as described in the literature, found that it is difficult to simultaneously deal with all the capacities of resilience, including absorption, adaptation, and recovery. Most existing methods lack the ability to cover all stages and include all capacities of resilience^38^. Thus, in order to bridge this gap in the existing research, a three-stage framework is proposed to periodically and dynamically evaluate the comprehensive resilience of RWSSs across different stages. Results are verified using a triangulation method, applying a quantitative analysis through PLS-SEM, combined with qualitative discussion of the latest literature.

### Research method

#### Proposing a theoretical framework

**Figure 1:**
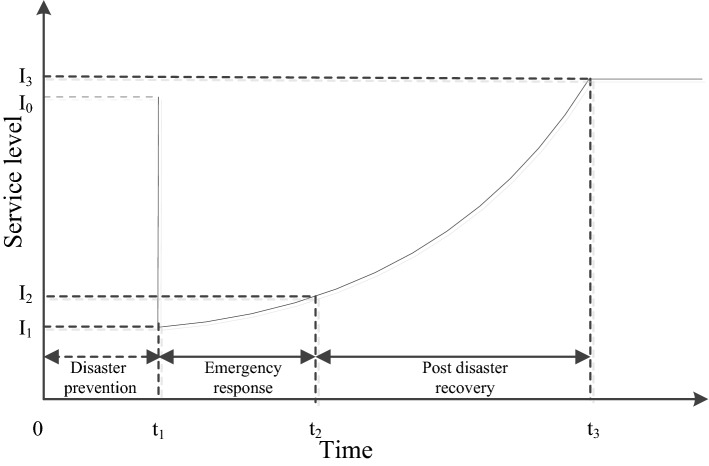
Response curve of RWSS after earthquake disaster.

As previously mentioned, the first step of this study is to establish a theoretical framework to prove the definition of resilience and its components. As a summary of the definition of resilience in the literature, the resilience of RWSSs in this study is defined as the absorption capacity to resist and absorb external interference in the disaster preparation stage, the adaptive capacity in the emergency response stage, and the capacity to quickly recover to an acceptable level in the post disaster recovery stage. On this basis, the seismic-resilience is represented by the service change of RWSS in the whole earthquake disaster management cycle based on previous research^6,9,10,30^, as shown in Fig. 1.

The first stage (0–t_1_) is the disaster prevention stage before earthquake, which is the normal state of the system. This stage mainly reflects the ability of the system to resist external interference and absorb the damage of earthquake within the seismic fortification level and other natural disasters to maintain the water supply service at I_0_.

The system enters into the emergency response stage (t_1_–t_2_) after an earthquake has occurred (t_1_). At this stage, the water supply service of the system rapidly dropped to I_1_ due to the damage of the water supply system caused by the earthquake. The water supply service capacity is improved to I_2_ by taking the emergency water supply and other measures to make up for the basic needs of the local water supply. This stage mainly reflects the ability of the system to adapt to the minimum water demand.

The third stage is the post-disaster recovery stage (t_2_–t_3_). This stage mainly reflects the recovery capacity of water supply services to an acceptable level (better than/equal to/lower than the service level before the earthquake) as soon as possible through the allocation of resources. The reconstructed system of the areas with weak disaster resistance should not only be restored to the level before the disaster but also need to exceed the previous level to improve the disaster resistance capacity of the system^8^. In addition to destruction, disasters provide an opportunity to improve the living conditions of victims through an effective reconstruction process^39^. Specifically, reconstruction is for the better^25,40^, especially in rural areas. Thus, the system service capacity I_3_ after reconstruction is higher than I_0_.

**Figure 2:**
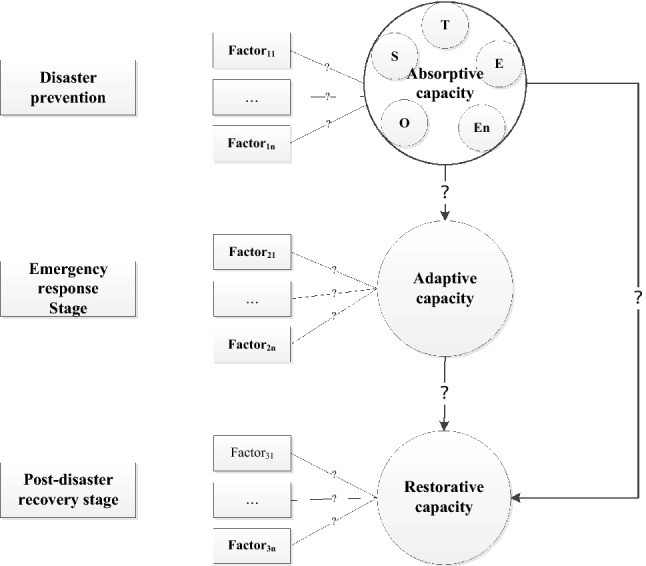
Three-stage framework for the seismic-resilience measurement of RWSSs.

Thus, a three-stage seismic resilience evaluation framework is proposed as the conceptual basis in this study according to the above three-stage characteristics of RWSSs, as shown in Fig. 2.

#### Identifying the factors of seismic-resilience

**Figure 3:**
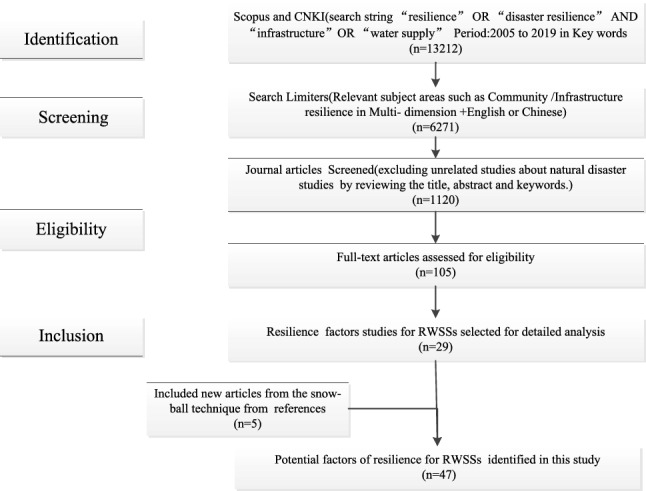
Identification process of the potential resilience factors for RWSSs.

The seismic resilience of the water supply system is a function of the system’s ability to deal with potential events^6,28^. The relationships set in the conceptual framework in Fig. 2 are used to describe the gap between resilience and factors at each stage. Only when relationships between factors are determined can the membership function between stages and factors be assigned. This study identifies potential influencing factors through four steps: Identification, Screening, Eligibility and Inclusion, which is a common method to guide the systematic review of academic literature^25^, as shown in Fig. 3. At the identification stage, articles were searched using a search string “resilience” OR “disaster resilience” AND “infrastructure” OR “water supply” as the key words published during 2005–2019 in the databases of Scopus in English and CNKI in Chinese. This search resulted in 13,212 articles, which were further reduced to 6271 articles in stage 2: screening, using further limiters, such as “relevant subject areas and language’’. In stage 3: Eligibility, 1120 articles were obtained for further screening by excluding articles irrelevant to natural disaster studies; then, 105 articles were further screened for a full-text review based on the title and abstract review. In stage 4: Inclusion, 47 initial potential factors were identified from 34 studies derived by screening the full text of the 105 eligible manuscripts and 5 articles from the references for further analysis.

The preliminary factors obtained through literature review are not suitable for data collection and factor analysis without adjustment. This is due to the different research purposes for which they were applied. Therefore, a semi-structured interview was conducted in which experts with rich knowledge of the field were solicited in order to consolidate factors obtained from literature^41^. Accordingly, 47 initial factors were screened to ensure that factors retained remained relevant to the evaluation of the seismic resilience of RWSSs. From July to August 2020, ten experts from public, private, and research institutions with at least 5 years of experience relevant to RWSSs, and who had participated in at least one earthquake relief effort regarding RWSSs voluntarily took part in the interview. This study conducted repeated interviews with experts to ensure the robustness of factor screening. The experts' opinions on the modification of initial factors were collected through the first round interviews. Before modifying the factors (delete/add/modify), a further round was conducted. Where greater than 70% of experts agreed with the modification^7^, the modification was implemented.

Experts revised the indicators using a range of criteria: (1) ‘Delete inapplicable indicators.’ Due to the spatial difference of toughness, some indicators that are not suitable for evaluating the seismic resilience of RWSSs have been deleted, such as "GDP". (2) ‘Merge redundant indicators.’ Here, such indicators as "social trust", "trust in the army" and "trust in rescue" are merged into "social trust". (3) ‘Modify the indicators in accordance with the characteristics of rural areas.’ For example, "water quality" was replaced by "environmental pollution". In recent years, the pollution problems in rural areas such as factory pollution and poultry pollution have become prominent. Experts believed that reference to environmental pollution more clearly expresses the impact of the environment on the water quality of rural water supply systems after an earthquake. The results were used to improve the indicators derived from previous research for evaluating the seismic resilience of RWSSs. Through several rounds of repeated modification, a final list of 41 factors affecting the seismic resilience of RWSSs were retrieved. See Table 2.

**Table 2:** Factors and groupings affecting the seismic-resilience of RWSSs.

Factor code	Factor name	Group	Source of references
CF01	Alternative water source	TRIDPS	^6,8,36,46^
CF02	Seismic design	TRIDPS	^6,8,37,47,48^
CF03	Emergency power	TRIDPS	^8,46^
CF04	Independent fire-water design	TRIDPS	^3,8^
CF06	Earthquake early warning system	TRIDPS	^35,47^
CF12	Proactive posture	ORIDPS	^7,49^
CF13	Effective partnership	ORIDPS	^7,47,49,50^
CF17	Laws and policies	ORIDPS	^7,51,52^
CF18	Organizational structure	ORIDPS	^7,8^
CF35	Earthquake intensity	ORIDPS	^46,48^
CF22	Social participation	ERIDPS	^36,37,41,47^
CF28	Available financial resources	ERIDPS	^7,8,35^
CF29	Gross regional product (GRP)	ERIDPS	^7,8,35^
CF30	Fast financing access	ERIDPS	^7,8,35^
CF31	Employment rate	ERIDPS	^6,7,51,53^
CF32	Operation and maintenance funds	ERIDPS	^46,54^
CF33	Periodic asset assessment	ERIDPS	^7,54^
CF41	Reconstruction model	ERIDPS	^40,46^
CF19	Cultural level	SRIDPS	^41,47,54^
CF21	Community publicity	SRIDPS	^6,7,36,51^
CF24	Place attachment	SRIDPS	^7,8,46,47^
CF25	Social trust	SRIDPS	^41,47^
CF26	Household water reserve	SRIDPS	^46,54^
CF34	Groundwater stock	EnRIDPS	^46,51^
CF36	Earthquake history	EnRIDPS	^46,53,55^
CF37	The time of the earthquake	EnRIDPS	^6,46^
CF38	Topographic	EnRIDPS	^10,53,56^
CF39	Climate conditions	EnRIDPS	^51,53^
CF40	Environmental pollution	EnRIDPS	^51,57^
CF07	Remaining service capacity	Adaptive capacity	^8,36,58^
CF09	Intelligent design	Adaptive capacity	^36,46^
CF11	Emergency response plan (ERP)	Adaptive capacity	^6,7,46,54,58^
CF14	Leadership	Adaptive capacity	^7,47,49^
CF20	Post disaster water demand	Adaptive capacity	^6,46,58^
CF27	Emergency water supply	Adaptive capacity	^7,58^
CF05	Professional reserve	Restorative capacity	^7,41,47^
CF08	Degree of system recovery	Restorative capacity	^8,46^
CF10	Maintenance information	Restorative capacity	^46,54^
CF15	Decision-making	Restorative capacity	^7,49^
CF16	Political will	Restorative capacity	^3,52^
CF23	Crisis insight	Restorative capacity	^41,47^

*TRIDPS: Technical resilience in the disaster prevention stage; ORIDPS: Organizational resilience in the disaster prevention stage; ERIDPS: Economic resilience in the disaster prevention stage; EnRIDPS: Environmental resilience in the disaster prevention stage.

#### Questionnaire survey

A questionnaire survey, a systematic method of collecting data based on interviewees' knowledge, is widely used in disaster management research to collect professional views^42–44^. In this study, the data used to verify the relationship between factors were obtained through the implementation of a questionnaire survey. The stakeholders of RWSSs (including emergency management official, design and planning personnel of RWSSs, and operation managers of RWSSs) were invited to comment on the questionnaire. Data have been collected by an online structured questionnaire, which is made according to the 41 factors (Table 2). The questionnaire consists of three different parts: Sect. 1 describes the objectives and confidentiality commitments, Sect. 2 collects general information about respondents, and Sect. 3 tests the importance of the 41 factors relative to the seismic resilience of RWSS. A five point Likert design is used to set standardized questions for each factor. The respondents were asked to score the importance of factors affecting the seismic resilience of RWSSs using "1–5", where 1 indicates particularly unimportant and 5 indicates particularly important. Taking "Alternative water source" as an example, the standardization problem is posed as "the importance of alternative water source to the seismic resilience of rural water supply systems".

Sichuan Province is one of the most earthquake-prone areas in China. Many devastating earthquakes that occurred in Sichuan brought great damage to local RWSSs, such as the Wenchuan earthquake in 2008 and the Lushan earthquake in 2013 (Table 1). In this study, the RWSS in earthquake prone areas of Sichuan Province is selected as the research object. Then, 1296 RWSSs near the earthquake area are focused, according to the list of RWSSs released by Sichuan Provincial Water Resources Department^45^. Finally, the RWSSs near the earthquake zone were divided into four regions (East, West, North, and South) according to the opinions of evaluation experts to further narrow the sample scope to 300 RWSSs. The questionnaires were issued during September 2020 to February 2021 considering the difficulty of obtaining rural data and the geographical differences of the rural areas.

#### Statistical analysis

To determine the relationship among factors and the seismic resilience of RWSSs in each stage, the principal component analysis (PCA), which is recommended in the literature for factor grouping^41^, was adopted to categorize identified factors into groups as the first step in this study. These groups of factors reflected the key aspects considered by managers of RWSS when evaluating system resilience. However, the resilience state of each stage may not be reflected by these groups, with the influence mechanism of resilience (the relationship between each factors group) also unspecified. To resolve this situation, groups of factors with hypothesized relationships were proposed to explain the resilience ability in different stages previously identified. Meanwhile, structural equation modeling (SEM) was utilized to test and validate these hypothesized relationships.

SEM is regarded as one of the most robust statistic techniques capable of analyzing complex interrelationships among variables in management science^44,59^. SEM can be undertaken as covariance-based SEM (CB-SEM) or partial least squares SEM (PLS-SEM) according to different analysis methods. Although both types are popular in academic studies^60^, PLS-SEM can effectively process small sample data without assuming the data distribution^44,61^. Thus, PLS-SEM was employed in this study in consideration of the characteristics of the data used.

The assessment of PLS-SEM includes two sequential steps, where the reliability of the measurement model is evaluated first. The measurement models are divided into reflection model (RM) and formation model (FM) according to the relationship between the observed variables (indicators) and the potential variables (structure). The type of measurement model must be determined before evaluation because the evaluation methods of the two models are different. The relationship between structure (factor group) and indicators (factors) is determined according to the factor analysis in this study, and the selected group represents the importance of the factors around it. According to the rules proposed by Hair et al.^62^, the measurement model in this study is RM. Four criteria are used to evaluate the reliability of RMs^44,62^: (1) Individual indicator reliability, (2) internal consistency reliability, (3) convergent validity, and (4) discriminant validity. The reliability of individual indicators reflects the correlation between indicators and their subordinate structure. The high load on the structure indicates that the indicators in the structure have a higher correlation. The recommended value of 0.7 was used as the reference threshold for adjustment to minimize the error^51^. The composite reliability (CR) is undertaken to evaluate the internal consistency reliability, whose value should be higher than 0.70^62^. The average variance extracted (AVE) is used to establish the convergent validity at the construct level^44,60^, and it’s value must be higher than 0.5, indicating that the structure explains more than half of the variance of the indicators^44,60,62^. The Fornell Larker criterion is an effective method for analyzing the discriminant validity^44^. The square root of AVE for each structure should be greater than its highest correlation with any other structure^61,62^. Then, the Bootstrapping technique is adopted to assess the significance of the path coefficients by SmartPLS 3.2.9, which is the common way to evaluate the structural model^61^. In this study, the number of bootstraps was set as 5000. Meanwhile, the number of cases was set as the number of effective questionnaires collected. In addition, the critical T-value for a two-tailed test was set as 1.65 (significance level at 10%), due to the exploratory nature of this study^44^.

## Results

### Sample characteristics

A total of 123 valid questionnaires were received, with the effective response rate of 41%, which was reasonable considering the normal rate of response in the disaster risk management studies^44,63,64^ The details of the respondents are summarized in Table 3. More than 80% of the respondents had more than 5 years of relevant experience. Approximately 78.86% of the respondents were operation managers of RWSSs, and 76.42% of them have experience in earthquake relief. Overall, the respondents can appropriately represent the opinions from the perspective of RWSS’s operation managers, and the sample obtained from the survey is robust.

**Table 3:** Summary of respondents' profile ( adapted from Wenmei et al.^2^).

Category	Frequency	%
**Field of work**		
Others (Designer/emergency management officer/planer)	26	21.14%
Operation management officer	97	78.86%
**Experience (years)**		
< 5	23	18.70%
5–10	34	27.64%
10–15	36	29.27%
> 15	30	24.39%
**Times of participating in earthquake relief of RWSSs**		
No relevant experience	29	23.58%
≥ 1	94	76.42%

### Results of factor analysis

The Kaiser–Meyer–Olkin score was 0.903, which is greater than the recommended threshold value of 0.6^65^. Meanwhile, the Bartlett test of Sphericity was also significant (0.000 < 0.05), indicating that the data obtained were suitable for the factor analysis. With the application of principal component analysis and maximum variance method with Kaiser normalization, nine principal components were extracted, carrying a factor loading of more than 0.5. An adjustment was applied because some component factors were slightly inconsistent in content, with the final set of factors classified into seven groups: Technical Resilience In the Disaster Prevention Stage (TRIDPS); Economic Resilience In the Disaster Prevention Stage (ERIDPS); Environmental Resilience In the Disaster Prevention Stage (EnRIDPS); Organizational Resilience In the Disaster Prevention Stage (ORIDPS); Social Resilience In the Disaster Prevention Stage (SRIDPS); Adaptive capacity, and Restorative capacity, as shown in Table 2.

According to the theoretical framework of hypothesis, a set of hypothetical were proposed to describe the relationship between factor groups and between factor groups and resilience, as shown in Table 4. The normality of the obtained data was examined by the Kolmogorov Smirnov test, where all the P-values were less than 0.05, indicating that the data were subjected to a non-normal distribution^59^. Therefore, PLS-SEM was clearly recommended to test and validate these hypothesized relationships. According to Cohen’s research, when the maximum number of independent variables in the model is 4, it needs at least 53 observations to achieve 80% statistical power to detect R^2^ values of at least 0.25 within a statistical probability error of 10%^62^. Therefore, the sample size of 123 in this study was more than sufficient for further data analysis using the PLS-SEM model.

**Table 4:** Seismic-resilience in three stages and the hypothetical relationships.

Stages	Hypothesized relationships	Remarks
Disaster prevention	ERIDPS → TRIDPS ORIDPS → TRIDPS SRIDPS → TRIDPS EnRIDPS → TRIDPS ERIDPS → ORIDPS ERIDPS → SRIDPS ERIDPS → EnRIDPS EnRIDPS → SRIDPS EnRIDPS → ORIDPS ORIDPS → SRIDPS	The impact of economic, social, environmental and organizational dimensions on system physical vulnerability, and the mutual restrictive relationship among economic, social, environmental and organizational dimensions
Emergency response	TRIDPS → Adaptive capacity	The influence of absorptive capacity in disaster preparedness stage on Adaptability in emergency response stage
Post-disaster recovery	TRIDPS → Restorative capacity Adaptive capacity → Restorative capacity	The influence of the absorptive capacity in disaster preparedness stage and the adaptability of emergency response stage on the system quick recovery capacity after earthquake

### Evaluating the measurement models

After the assumption of the initial model is completed, it needs to be iteratively modified and analyzed to form the most suitable theoretical model for data collection and support. The reliability of the initial measurement model is evaluated according to the four criteria of RMs, and the results are shown in Tables 5 and 6. The results showed that the loading of the five factors were lower than the recommended reference value of 0.7^44^ (CF01 = 0.683; CF08 = 0.699; CF19 = 0.646; CF29 = 0.647; CF33 = 0.652). The potential practical significance of these indicators needs to be considered before removal^44^. All factors were arranged in a descending order based on the relative importance by calculating the average and standard deviation, in which CF01 ranked 2th, CF08 ranked fifth, CF19 ranked 40th, CF29 ranked 33rd, and CF33 ranked 41st. According to Pareto’s principle, the top 20% of the ranking factors are taken as critical factors (8/41). Consequently, CF01 and CF08 were retained, while the other three measurement variables were dropped before accepting the final structural model. Furthermore, the AVE and CR values all appeared above the thresholds (AVE > 0.5, CR > 0.7)^64^, as presented in Table 5. Finally, the results of the discriminant validity shown in Table 6 show no correlation between any two groups of factors, indicating that the factor groups are different from each other. Thus, the measurement models were reliable and valid, allowing for the following structural model evaluation.

**Table 5:** Measurement model evaluation result.

Constructs	Factor code	Loading	Cronbach's alpha	Composite reliability (CR)	Average variance extracted (AVE)
TRIDPS	CF01	0.682	0.825	0.877	0.589
	CF02	0.755			
	CF03	0.862			
	CF04	0.726			
	CF06	0.799			
ORIDPS	CF12	0.807	0.858	0.898	0.639
	CF13	0.714			
	CF17	0.807			
	CF18	0.823			
	CF35	0.840			
ERIDPS	CF22	0.821	0.879	0.909	0.624
	CF28	0.837			
	CF30	0.798			
	CF31	0.702			
	CF32	0.783			
	CF41	0.795			
SRIDPS	CF21	0.774	0.767	0.851	0.589
	CF24	0.827			
	CF25	0.752			
	CF26	0.712			
EnRIDPS	CF34	0.722	0.858	0.894	0.584
	CF36	0.754			
	CF37	0.773			
	CF38	0.735			
	CF39	0.756			
	CF40	0.841			
Adaptive capacity	CF07	0.796	0.878	0.908	0.625
	CF09	0.806			
	CF11	0.702			
	CF14	0.726			
	CF20	0.805			
	CF27	0.892			
Restorative capacity	CF05	0.823	0.887	0.851	0.589
	CF08	0.699			
	CF10	0.783			
	CF15	0.814			
	CF16	0.805			
	CF23	0.868

**Table 6:** Fornell–Larcker criterion.

	Adaptive capacity	ERIDPS	EnRIDPS	ORIDPS	Restorative capacity	SRIDPS	TRIDPS
Adaptive capacity	**0.790**						
ERIDPS	0.733	**0.790**					
EnRIDPS	0.721	0.627	**0.764**				
ORIDPS	0.740	0.774	0.749	**0.799**			
Restorative capacity	0.719	0.748	0.593	0.764	**0.800**		
SRIDPS	0.649	0.698	0.652	0.655	0.684	**0.767**	
TRIDPS	0.703	0.596	0.693	0.680	0.699	0.671	**0.767**

The diagonal elements (in bold) are the square root of the AVEs; non-diagonal elements are latent variable correlations.

### Evaluating structural models

**Figure 4:**
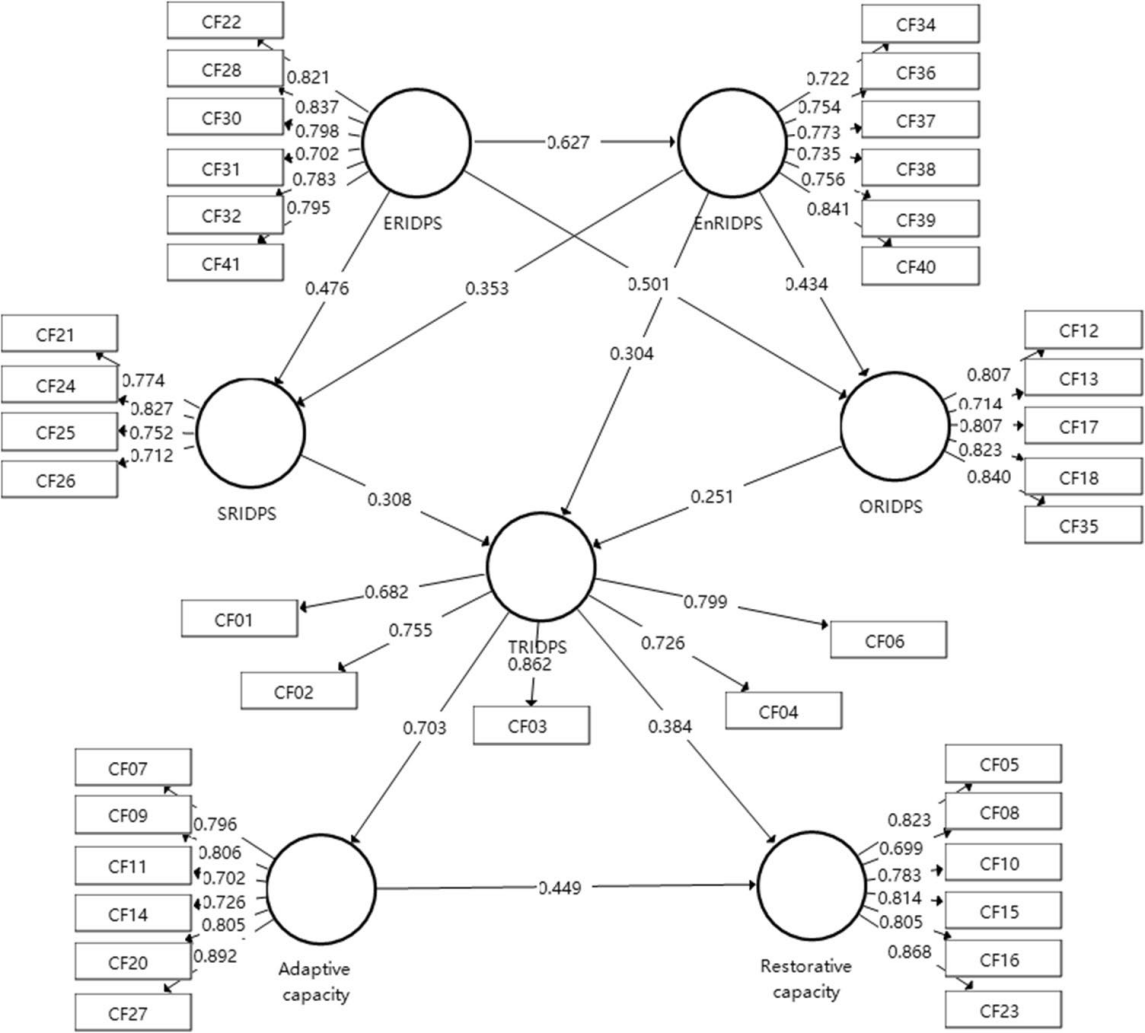
Final relationship model for seismic-resilience of RWSSs.

All paths and their coefficients between groupings of factors determined by bootstrapping in the initial model were summarized in Table 7. Four pathways were not significant at the level of 0.10 (T < 1.65), which were deleted in a descending order according to the size of the T value^62^. Finally, 11 of the 13 paths are verified, and the final model of the relationship between the factors affecting the seismic resilience of RWSS is determined, as shown in Fig. 4.

**Table 7:** Path coefficients and significance of the initial model.

Relation (hypothesis)	Path coefficient	T-value	Inference
Adaptive capacity → Restorative capacity	0.448	3.465	Support
TRIDPS → Adaptive capacity	0.703	12.209	Support
TRIDPS → Restorative capacity	0.385	2.956	Support
EnRIDPS → TRIDPS	0328	2.317	Support
EnRIDPS → SRIDPS	0.391	2.836	Support
EnRIDPS → ORIDPS	0.436	4.769	Support
SRIDPS → TRIDPS	0.436	1.524	Not support
ORIDPS → SRIDPS	0.155	1.523	Not support
ORIDPS → TRIDPS	0.251	0.960	Not support
ERIDPS → TRIDPS	0.062	0.373	Not support
ERIDPS → EnRIDPS	0.627	10.552	Support
ERIDPS → SRIDPS	0.350	3.123	Support
ERIDPS → ORIDPS	0.501	4.766	Support

The indirect effect and total effect between each factor group were calculated according to the final verified model to build the seismic resilience evaluation model of RWSS, as shown in Table 8. There were 20 paths in the structural model, including seven direct path coefficients, nine indirect paths, and four paths with direct and indirect effects. According to the variance explanation, the VAF of these four paths ranges from 20 to 80%, indicating that part of the regulation occurs in these four paths^62^.

**Table 8:** Path coefficient among construct.

Path	Path coefficient	Total indirect effects	T-value	Total effects	T-value	VAF (%)
ERIDPS → EnRIDPS	0.627	–	–	0.627	10.803*	–
ERIDPS → SRIDPS	0.476	0.221	3.225*	0.697	9.187*	40.22%
ERIDPS → TRIDPS	–	0.6	11.894*	0.6	11.894*	100%
ERIDPS → ORIDPS	0.501	0.272	3.766*	0.773	8.474*	35.19%
ERIDPS → Adaptive capacity	–	0.367	5.789*	0.367	5.789*	100%
ERIDPS → Restorative capacity	–	0.403	6.510*	0.403	6.510*	100%
EnRIDPS → SRIDPS	0.353	–	–	0.353	3.607*	–
EnRIDPS → ORIDPS	0.434	–	–	0.434	4.577*	–
EnRIDPS → TRIDPS	0.304	0.218	3.319*	0.522	5.785*	41.72%
EnRIDPS → Adaptive capacity	–	0.367	5.789*	0.367	5.789*	100%
EnRIDPS → Restorative capacity	–	0.365	6.314*	0.365	6.314*	100%
ORIDPS → TRIDPS	0.251	–	–	0.251	1.964*	–
ORIDPS → Adaptive capacity	–	0.176	1.899*	0.176	1.899*	100%
ORIDPS → Restorative capacity	–	0.175	1.862*	0.175	1.862*	100%
SRIDPS → TRIDPS	0.308	–	–	0.308	2.672*	–
SRIDPS → Adaptive capacity	–	0.217	2.508*	0.217	2.508*	100%
SRIDPS → Restorative capacity	–	0.215	2.435*	0.215	2.435*	100%
TRIDPS → Adaptive capacity	0.703	–	–	0.703	12.225*	–
TRIDPS → Restorative capacity	0.384	0.315	3.712*	0.699	6.751*	45.06%
Adaptive capacity → Restorative capacity	0.449	–	–	0.449	3.553*	–

*Significance level at 0.1.

## Discussion

### Path analysis of economic resilience in disaster preparedness stage

The findings indicated that economic resilience in the disaster prevention stage (ERIDPS), as measured by social participation (CF22), available financial resources (CF28), fast financing access (CF30), employment rate (CF31), operation and maintenance funds (CF32), and reconstruction model (CF41), has the most extensive effect on the other six structures. This finding is consistent with the conclusions in the literature that economic factors play a significant role, directly and indirectly affecting the other factors^35,66^. The existence of rural water supply infrastructure does not mean that local residents have access to safe and reliable drinking water^1,67,68^, The operation, maintenance, and finance of rural water supply infrastructure may be in a suboptimal position^1,69^, which will threaten the resilience of the water supply system; for example, the water supply system in Kathmandu was inefficient before the earthquake due to the lack of operation and maintenance funds^46^. The social participation indicates the willingness of peoples’ involvement in communities, which is measured by the world giving index^41^. Therefore, this parameter is also one of the important economic factors in the disaster prevention stage. The reconstruction model has a great influence on post disaster recovery. Traditionally, a donation-driven model was thought to be the fastest and most effective way of post-disaster reconstruction^70^. However, the owner-driven model is regarded a better way, considering the long-term disaster resilience^40^. In China, different reconstruction models mean various financial allocations. Given the great threat of earthquake, the Chinese government has been exploring post-disaster reconstruction models to improve economic resilience. For example, the reconstruction cost of Wenchuan was mainly allocated by the central government according to the post-disaster reconstruction planning because the reconstruction was led by the state^71^. Meanwhile, the Sichuan provincial government was mainly responsible for the funds of Lushan post-earthquake reconstruction^72^. The local government of Aba was mainly responsible for the funds for post-earthquake reconstruction of Jiuzhaigou^73^. In addition, factors, such as available financial resources, fast financing access, and employment rate, are regarded as important factors that affect the post-disaster recovery capacity of UWSS^6,8^. Available financial resources, fast financing access, employment rate, and reconstruction model are considered to be more closely related to the post-disaster recovery stage. However, from a dynamic point of view, considering these factors in the disaster preparedness stage is more conducive to learn from past disaster events and formulate a better resilience strategy because communities are always between two disasters (past and future), and resilience to past disasters affects community resilience to future disasters^25^.

### Path analysis of environmental resilience in disaster preparedness stage

The paths of environmental resilience in the disaster prevention stage (EnRIDPS), as measured by groundwater stock (CF34), earthquake history (CF36), the time of the earthquake (CF37), topography (CF38), climate conditions (CF39), and environmental pollution (CF40), show that the EnRIDPS has a significant effect on the technical, social, and organizational aspects of RWSSs. Environmental dimension is of great significance to water supply system due to the spatial–temporal characteristics of system resilience^6^. Some water supply systems in Kathmandu faced groundwater depletion before the earthquake, resulting in the failure of household water reserve, such as wells and pumps, to obtain enough water^46^. In addition, topography is the one of main factors that cause the social and economic toughness of mountainous areas to be lower than that of plain areas in Taiwan^56^. These conclusions are consistent with the effect of the environment effect on the social dimension in this study. The effect of the environment on the technical dimension is evident. For example, hot weather will increase post-disaster water demand (including residential water and fire water)^6^, which means that the climate conditions where the RWSSs is located affect the fire–water design to a certain extent. Destructive earthquakes will inevitably occur in Kathmandu Valley according to the local earthquake history^46^. In addition, the Chinese government promulgated the law “the People’s Republic of China on earthquake prevention and disaster reduction”, which stipulates the seismic design requirements of infrastructure, to cope with the damage to infrastructure and communities caused by earthquakes^74^. These conclusions are consistent with the effect of the environment on the organizational factor groups (including seismic intensity and law and policy) in this study.

### Path analysis of organizational and social resilience in disaster preparedness stage

The paths of organizational resilience and social resilience in disaster prevention stage show that they have a significant impact on technical resilience. Meanwhile, the relationship between organizational and social dimensions is not significant. Organizational resilience is considered to be a key dimension in assessing the resilience of community^8^ and various infrastructures^6,75^. In this study, organizational structure, laws and policies, earthquake intensity, effective partnership, and proactive posture were identified as organizational resilience factors in the disaster preparedness stage. The influence of organizational dimension on the technical dimension is obvious. As previously mentioned, the seismic design of RWSS needs to meet the corresponding seismic design laws and regulations. In addition, the earthquake intensity affects the development of earthquake early warning system. For example, Wenchuan County became the first county in China to realize a multi-disaster early warning service system due to the super destructiveness of Wenchuan M8.1 earthquake^76^. Social trust, local attachment, household water reserve, and community publicity were identified as social resilience factors in the disaster preparedness stage. Social dimension is also an important factor that affects the seismic resilience of a water supply system^7,12^. The time of system recovery cannot be effectively estimated without considering the influence of social dimension^6^. In disaster prone areas, water supply companies encourage people to keep enough household water reserve for 7 days or more. Accordingly, repair personnel have the opportunity to use better repair technology rather than faster repair technology, such as use pipelines with better seismic performance rather than pipelines as fragile as before the earthquake^41^, which will affect the future resilience of the water supply system to withstand earthquake disasters next time. This finding is consistent with the effect of social dimension on technical dimension in this study.

### Path analysis of technical resilience in disaster preparedness stage

The paths of technical resilience in the disaster prevention stage (TRIDPS), which is composed of alternative water source, seismic design, emergency power, independent fire–water design, and earthquake early warning system, indicated the comprehensive influence of non-technical dimensions on the physical vulnerability of RWSSs. The previous analysis discussed the effect of different non-technical dimensions on the technical dimension alone. The technical resilience in the disaster preparedness stage is comprehensively affected by other dimensions. Accordingly, the absorption capacity is developed in the disaster preparedness stage. For example, the design of a water network is limited by economic, geographical, and other environmental conditions^41^. In addition, budget, construction, and transportation constraints make it ineffective to actually strengthen water supply under various geographical conditions (such as remote rural areas)^37^.

### Path analysis of adaptability and post-disaster recovery ability

The paths of adaptability and post-disaster recovery ability reflected the continuous process of RWSSs responding to earthquake, and the system resilience level of the subsequent stage is affected by the resilience level of the previous stage, which is consistent with the research conclusions in the literature^6,8^. When the absorptive capacity of the system is insufficient to cope with the consequences of the disaster (in case of destructive earthquake), the adaptability of the system will minimize the adverse consequences^6^. If the impact of the damage event on the system does not exceed the absorption capacity, then the system will quickly serve without affecting the service, and the system’s recovery capacity will be maintained at the maximum level. If the damage is between the absorption capacity and the recovery capacity of the system, then the system may need medium and long-term recovery. If the damage of the system exceeds the absorption and adaptability of the system, then it will take a long time or even international assistance to recover^6^.

**Figure 5:**
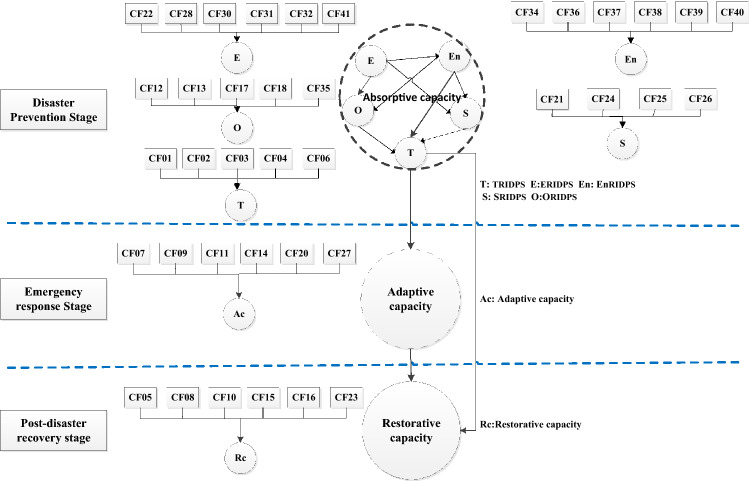
Final empirical evaluation framework of seismic-resilience for RWSSs.

According to the verified models and paths, the seismic capacity of RWSSs is a continuous dynamic process, and it can be measured as: the absorptive capacity in the disaster prevention stage, which is the result of the comprehensive influence of social, environmental, organizational, and economic on the technical dimension; the adaptive capacity in the emergency response stage, which is the result of the comprehensive influence of various emergency measures coupled with the absorptive capacity in the disaster prevention stage; and the recovery capacity in the post-disaster recovery stage, which is result of the absorptive and adaptive capacity coupled with the implementation of the recovery measures. Thus, a three-stage dynamic evaluation framework for seismic resilience of RWSSs is developed, as shown in Fig. 5.

## Conclusion

This work found that economic and environmental resilience in the disaster preparedness stage are the two most important dimensions that affect the seismic resilience of RWSS, as shown in Fig. 5. The resilience measurement is a complex systems engineering because resilience is affected by interrelated multidimensional factors. With the understanding of the dynamic characteristics of resilience, recent studies emphasize the importance of studying the dynamically relationship between different dimensional factors of resilience for resilience measurement and improvement^35,77^. In this study, a three-stage dynamic evaluation framework for measuring the seismic resilience of RWSSs is first proposed. Then, the potential factors are determined through literature review, and the factors are modified through expert interviews. Data were collected through questionnaires. Finally, the relationship between various factors and the potential impact mechanism of seismic resilience for RWSS are demonstrated by triangular analysis (quantitative data analysis and qualitative literature analysis). Awareness of these factors and their influence on seismic-resilience of RWSSSs will enable local authorities to identify existing strengths and weaknesses with regard to these factors.

Furthermore, evaluating the seismic resilience of RWSSs by stages is an additional strength of this approach. Given the continuing threat earthquakes pose to water supply systems, the seismic resilience of water supply systems must be assessed^78^. However, multi-dimensional factors influence the seismic resilience of RWSSs to varying degrees across event chronologies during the disaster management cycle. Accordingly, it is a great challenge to collect data across each stage at the same points in time for rural areas. This study linked each factor with the corresponding disaster preparedness, emergency response, and post-disaster recovery stages, verifying the relationship between multi-dimensional factors and revealing the potential influence mechanism of seismic resilience of RWSS through triangulation. Therefore the evaluation scope can be effectively reduced to the limited relevant factors at the current stage while evaluating the seismic resilience state at different stages of the disaster management cycle. This situation greatly reduces the evaluation workload, making it possible for local authorities to periodically evaluate resilience and form resilience basic data. However, the coexistence of qualitative and quantitative factors and the inevitable loss of information in disaster events need to be considered while measuring the seismic-resilience of RWSSs. Therefore, appropriate algorithms based on this research must be introduced in the next study to effectively deal with the problems of information fusion and information loss.

## Ethical considerations

The research ethics committee of Sichuan University has approved this study. The survey protocol adheres to all current laws of China, strictly complies with the academic ethics requirements of the association of Management Sciences of Sichuan University and Sichuan University-The HongKong Polytechnic University Institute for Disaster Management and Reconstruction, Informed consent was obtained from all participants at the time of data collection. Voluntary participation and right to withdraw at any time were assured.

## Data Availability

Data used and analyzed during this study is available from the corresponding author by request.
